#  Single nucleotide polymorphisms that differentiate two subpopulations of *Salmonella *enteritidis within phage type

**DOI:** 10.1186/1756-0500-4-369

**Published:** 2011-09-26

**Authors:** Jean Guard, Cesar A Morales, Paula Fedorka-Cray, Richard K Gast

**Affiliations:** 1 Agricultural Research Service (ARS), U. S. Department of Agriculture (USDA), 950 College Station Road, Athens, GA, 30605, USA

**Keywords:** Evolution, *Salmonella*, egg, chicken, genome, epidemiology

## Abstract

**Background:**

*Salmonella *Enteritidis is currently the world's leading cause of salmonellosis, in part because of its ability to contaminate the internal contents of eggs. Previous analyses have shown that it is an exceptionally clonal serotype, which nonetheless generates considerable phenotypic heterogeneity. Due to its clonality, whole genome analysis is required to find genetic determinants that contribute to strain heterogeneity of *Salmonella *Enteritidis. Comparative whole genome mutational mapping of two PT13a strains that varied in the ability to contaminate eggs and to form biofilm was achieved using a high-density tiling platform with primers designed from a PT4 reference genome. Confirmatory Sanger sequencing was used on each putative SNP identified by mutational mapping to confirm its presence and location as compared to the reference sequence. High coverage pyrosequencing was used as a supporting technology to review results.

**Results:**

A total of 250 confirmed SNPs were detected that differentiated the PT13a strains. From these 250 SNPS, 247 were in the chromosome and 3 were in the large virulence plasmid. SNPs ranged from single base pair substitutions to a deletion of 215 bp. A total of 15 SNPs (3 in egg-contaminating PT13a 21046 and 12 in biofilm forming PT13a 21027) altered coding sequences of 16 genes. Pyrosequencing of the two PT13a subpopulations detected 8.9% fewer SNPs than were detected by high-density tiling. Deletions and ribosomal gene differences were classes of SNPs not efficiently detected by pyrosequencing.

**Conclusions:**

These results increase knowledge of evolutionary trends within *Salmonella enterica *that impact the safety of the food supply. Results may also facilitate designing 2^nd ^generation vaccines, because gene targets were identified that differentiate subpopulations with variant phenotypes. High-throughput genome sequencing platforms should be assessed for the ability to detect classes of SNPs equivalently, because each platform has different advantages and limits of detection.

## Background

*Salmonella enterica *subspecies I serotype Enteritidis (*S*. Enteritidis) is a leading cause of salmonellosis worldwide [1,2]. It is the only serotype of approximately 1400 that has evolved the ability to survive in the internal contents of eggs produced by otherwise healthy hens and to be linked to frequent human illness [3]. *S*. Enteritidis contaminates foods other than eggs and it colonizes animals other than chickens [2,4,5]. However, it is predominantly associated with eggs, egg products, poultry, the farm environment, and cross contamination of other foods from eggs [1,3,4,6]. *S*. Gallinarum and *S*. Pullorum are avian-pathogenic serotypes of *Salmonella enterica *that are closely related to *S*. Enteritidis. They too contaminate eggs, but they have accumulated a number of pseudogenes that severely limit host range [7,8]. Other Salmonellae may be found in eggs, but only *S*. Enteritidis does so in a manner that propagates efficiently through the food chain. *S*. Enteritidis is unique in part because it produces a specialized LPS O-antigen capsule that contributes to long term survival in eggs [9-11].

Strains of *S*. Enteritidis vary greatly in their ability to contaminate eggs and this virulence attribute is independent of phage type lineage [12,5-15,10]. The ability to metabolize a wide range of amino acids has been linked to virulence [10,11,16], as has the ability to make the O-antigen capsule. Strain heterogeneity and variant metabolic profiles may facilitate completion of the infection pathway by overcoming a multitude of microenvironments resulting from cellular barriers, flock immunity and management practices [12]. Another reason *S*. Enteritidis may generate strain heterogeneity is to facilitate colonization of the reproductive tract of hens, which is subject to hormonally-dependent cyclical changes that impact local immunity and cell function [12,17,18]. *S*. Enteritidis produces 3 classes of fimbriae that contribute to colonization and invasion [19]. *S*. Enteritidis has a full repertoire of virulence factors in common with other pathogenic Salmonellae such as *S*. Typhimurium [8,20,21].

To understand more details of the evolutionary trends that contribute to the unique ability of some strains of *S*. Enteritidis to contaminate eggs requires analysis of whole genome databases, in large part to select genes relevant for evaluating in biological studies. We applied three methods of genome analysis to detect and confirm single nucleotide polymorphisms (SNPs) that differentiate gene content of two PT13a *S*. Enteritidis strains. These approaches were mutational mapping using a high density tiling microarray, pyrosequencing and confirmatory F/R Sanger sequencing of all suspect SNPs. A critical component of the research was strain selection. The strains that were compared were previously shown to vary in metabolic properties and in the ability to contaminate eggs [12,22,23]. Strain PT13a 21046 contaminates eggs, but does not form biofilm. In contrast, PT13a 21027 does not contaminate eggs, but does form biofilm. The phenotypes for these strains are thus BF+, EC- and BF-, EC+ respectively. Both of the PT13a strains were compared directly to strain PT4 22079, which contaminates eggs, forms biofilm (BF+, EC+) and has a metabolic profile intermediate to that of the two PT13a strains [11]. Thus, two highly clonal PT13a strains were available for comparing to a slightly more genetically distant strain of a different phage type. By triangulating three genomic databases, it became possible to find only those SNPs that differentiated the two PT13a strains. By using redundant methods for processing genomes, we increased the stringency of analysis and revealed some inherent limitations of each method. Results here show the chromosomal differences that differentiate two PT13a strains that vary in the ability to contaminate eggs and to form biofilm. This work was made possible by the availability of the whole genome sequence from PT4 *S*. Enteritidis P125109 (Refseq NC_011294) [8].

## Results

### Biofilm formation facilitated strains selection

**Figure 1:**
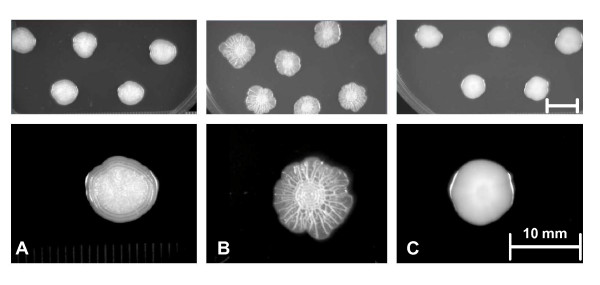
**Prominent colony morphologies of *Salmonella *Enteritidis**. TOP. Multiple colonies, 10 per plate, were grown for five days at ambient temperature following a 16 hr incubation at 37°C. BOTTOM. Colonies are magnified. Inset markers are 10 mm. This picture is reprinted with permission from *Avian Diseases *[12]. Copyright is property of the federal government. A. PT4 *S*. Enteritidis strain 22079, with weak slow biofilm formation; B. PT13a *S*. Enteritidis strain 21027, with strong rapid biofilm formation; C. PT13*a S*. Enteritidis strain 21046, with no discernible biofilm formation.

Biofilm formation was a useful phenotypic trait for identifying strains for comparison, because it was a simple agar-based assay that facilitated identification of strains that varied in metabolic properties, invasion potential, and in the ability to contaminate eggs (Figure 1) [17]. To reiterate, PT13a 21027 formed a strong biofilm but did not contaminate eggs (BF+ EC-), PT13a 21046 did not form biofilm but did contaminate eggs (BF- EC+), whereas PT4 22079 formed biofilm and contaminated eggs (BF+ EC+). The strong biofilm of PT13a 21027 was distinguishable from the weak one formed by PT4 22079, because the latter took longer to form and was never developed as in 21027 [Figure 1].

### *S*. Enteritidis strains chosen for analysis have commonly encountered PFGE patterns

**Figure 2:**
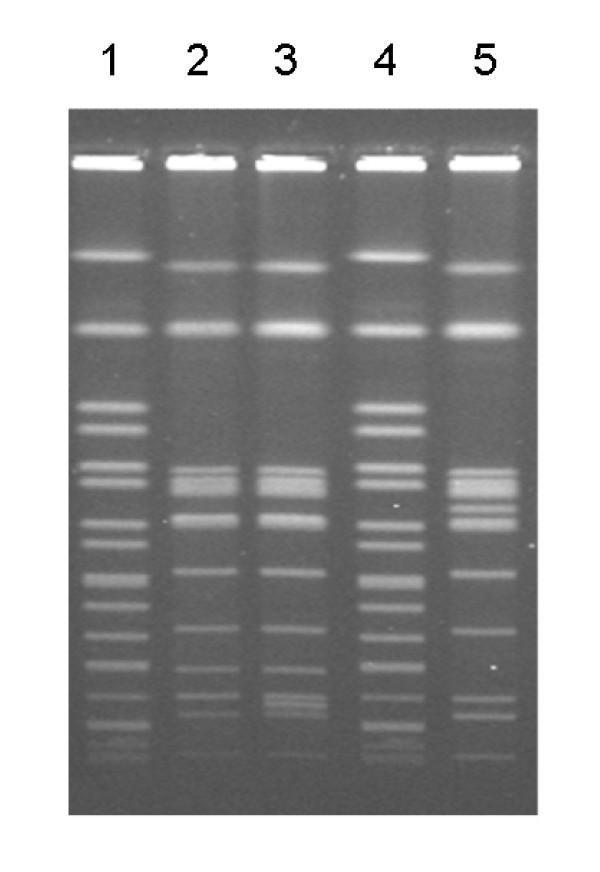
**PFGE patterns of *Salmonella *Enteritidis strains with known subpopulation characteristics**. Lanes 1 and 4, DNA fragment size markers; lane 2, PT13a *S*. Enteritidis strain 21027, which forms biofilm but does not contaminate eggs; lane 3, PT13a *S*. Enteritidis strain 21046, which does not form biofilm but does contaminate eggs; lane 5, PT4 *S*. Enteritidis strain 22079, which forms biofilm and contaminates eggs. *S*. Enteritidis strains 21027, 22079 and 21046 had the 1^st^, 6^th ^and 10^th ^most common PFGE profiles in the US, respectively, according to PulseNet typing classification schemes. Relative incidence may change by publication date.

At the time of analysis, the USDA VetNet PFGE database had 676 Enteritidis isolates and 51 unique patterns. PT13a 21027 was pattern JEGX01.0003 ARS (PulseNet equivalent pattern JEGX01.0004) (Figure 2). This pattern is the most common Enteritidis pattern in the database (308 out of 676 - 45.56%). PT13a 21046 was pattern JEGX01.0013 ARS (PulseNet equivalent pattern JEGX01.0037) (Figure 2). This pattern is the 10^th ^most common Enteritidis pattern in the database (3 out of 676 - 0.44%). PT4 22079 was pattern JEGX01.0017 ARS (PulseNet equivalent pattern JEGX01.0002) (Figure 2). This pattern is the 6^th ^most common Enteritidis pattern in the database. PFGE results indicate that the two strains being compared in reference to the PT4 genome are representative of strains found in outbreaks. PT13a 21027, which had the most common VN PFGE fingerprint, had a prominent biofilm within 48 hr at ambient temperatures on select media.

### Subpopulation biology of *S*. Enteritidis results from accumulation of SNPs, only some of which appear consequential for phenotype

Of the 250 confirmed polymorphisms that differentiated PT13a 21046 from PT13a 21027, 132 (52.8%) did not alter amino acid sequence (Table 1; For details, see Additional File 1). Of the 132 polymorphisms that did not alter amino acid sequences, 38 were intergenic, 12 involved ribosomal genes, and 82 were synonymous nucleotide substitutions The 3 SNPs that were in virulence plasmids were intergenic. Of the 250 polymorphisms, 115 (46.0%) were non-synonymous and thus they altered primary amino acid sequence. Five of 250 SNPs (2%) introduced premature termination codons in putative genes. Table 1 summarizes the types of SNPs detected. Overall, less than 0.01% of the total genome content differentiated PT13a subpopulations. However, when expressed as a percentage of total genes with non-synonymous changes, 1.1% and 1.5% of genes from PT13a 21046 and PT13a 21027 varied from PT4 22079, respectively. PT13a 21046 had a higher incidence of SNPs in ribosomal genes than did PT13a 21027, but PT13a 21027 accumulated more ORF-disrupting events than did PT13a 21046 (Table 1).

**Table 1:** Summary of 250 confirmed SNPs that differentiate subpopulations of *Salmonella enteritidis *PT13a

**Type of genetic change^a^**	**PT13a 21027**	**PT13a 21046**
		
Deletion^b^	8	2
Change in termination^c^	4	2
Amino acid substitution	55	44
RNA gene change	4	8
Intergenic polymorphism	22	19^c^
Synonymous	43	39
**TOTAL**	**136**	**114**

^a^SNPs composed of contiguous base pairs, ranging from doublets to a 215 bp deletion, are counted as single SNPs.

^b^A deletion in ydcZ changes one AA and deletes two others (counted as one DEL event).

^c^lncludes 3 SNPs in the virulence plasmid, which were all intergenic.

Detection of rare substitutions of amino acids within proteins is important, because a dramatic change in the class of amino acid is more likely to affect function. Of the 100 amino acid substitutions, neutral non-polar (nn) and neutral polar (np) amino acids were frequently substituted within the two chemical classes, but substitution between acidic and basic polar amino acids did not occur (Table 2). Between these two extremes, substitution of np for acidic (ap) or basic (bp) amino acids was very rare. Further research is needed to show the impact of altered amino acid sequence on protein function.

**Table 2:** Amino acid substitutions of *Salmonella enteritidis *PT13a

*substitution*	**Number in 21027**^**b**^	Number in 21046
ap to bp	0	0
bp to ap	0	0
np to ap	1	0
np to bp	1	0
ap to nn	2	2
nn to bp	2	3
bp to bp	2	1
np to np	2	2
ap to ap	3	1
ap to np	3	1
bp to nn	4	3
bp to np	4	5
nn to ap	4	1
nn to np	6	9
np to nn	7	4
nn to nn	15	12
**Total**	**56**	**44**

^a ^Classes of amino acids are: nn, neutral non-polar; np, neutral polar; ap, acidic polar; bp, basic polar. For greater detail about amino acid polarity refer to supplementary material (S1, because polar amino acids are not classified as weak or strong in this table.

^b^includes AA change in ydcZ, which also has a deletion.

### Lysogenic bacteriophage in the reference genome but absent in test genomes was easily detected

Mutational mapping of the two PT13a strains gave a prominent signal at a site in the reference genome known to include an ST64b-like bacteriophage, which defines the PT4 lineage. The absence of binding by the PT13a strains to the PT4 primers that hybridized ST64b-like sequence resulted in a run length (number of consecutive events) of more than 5500 consecutive mispriming events occurring in a window of approximately 47,000 bp. This large run of mispriming events has so far only been associated with the presence of a lysogenic bacteriophage.

### A bacteriophage absent in the reference but present in the test strains was detected by interval mapping

The strategy used for comparison of genomes is inherently biased against detection of inserts in PT13a genomes compared to the PT4 reference genome, because no PT13a specific primers would have been generated from the PT4 genome. For example, DNA-DNA microarray hybridization had previously detected bacteriophage Fels2 in *S*. Enteritidis PT13a strains [7,11]. However, there was no possibility of detecting bacteriophage Fels2 within PT13a strains by mutational mapping in the high-density tiling approach, because there was no template in *S*. Enteritidis PT4 for production of primers that would hybridize Fels2 sequence. To objectively assess whole genome data for the presence of sequence unique to test strains and absent in the reference, an interval map based on the average number of genes between SNPs was constructed from all data available in supplementary material (Additional File 2). Interval mapping of all SNPs from both strains against *S*. Typhimurium LT2 http://www.ncbi.nlm.nih.gov/nuccore/NC_003197) helped to locate the Fels2 bacteriophage in PT13a strains between *S*. Enteritidis gene (SEN) SEN2609 and SEN2624 of the PT4 genome. This location was confirmed by sequencing the beginning and end of the bacteriophage 3' to SEN2592 and 5' to SEN2623, respectively (data not shown); however, the complete bacteriophage sequence was not sequenced *de novo*. An additional benefit of interval mapping is that it appears to locate regions that have significant gaps in alignment defined by a gap of only 5 genes. This could help to locate even small inversions and gene transfers between serotypes. Otherwise, a strategy similar to interval mapping applied here is used elsewhere in software that depicts gene inversions and alignments in a graphing output (e. g. MAUVE); however, we needed finer detail to evaluate alignments to avoid missing inserted genes or non-coding sequence between the PT13a strains.

### Assay of raw data for deletion events can be used to detect multiple deletion events across the genome but may miss single nucleotide events

A useful feature of whole genome analysis that would facilitate outbreak investigations is if raw data could be quickly gleaned for evidence of mutational events that would distinguish strains within serotypes. For this reason, we analyzed technical details of the size of window and mispriming events (run length) associated in the raw data with SNPs that were confirmed as resulting from a deletion. The 215 bp deletion in STM4551 of PT13a 21046 occurred within a 231 bp window as reported in raw data files. It was associated with 34 mispriming events, or a run length of 34. The 92 bp deletion in STY3762 of PT13a 21046 had a run length of 14 within a 91 bp window. The 10 bp deletion within *dsdA*, the 12 bp deletion in *lrgB*, and the 12 bp deletion in *lysR *(STM2912), all of which occurred in PT13a 21027, each had a run length of 4 that spanned a 21 bp window. The 11 bp deletion that introduced the read-through fusion of *yjfK *and *yjfL *of PT13a 21027 had a run length of 6 and a 35 bp window. The smallest deletion was 1 run length (1 mispriming event), which was confirmed to be a 1 bp deletion in the gene *sefD *of PT13a 21027. In a run, each mispriming event is separated by 7 bp due to the nature of the high-density tiling approach and the size of the primer overlap. Raw data showed that in some cases there were easily observed spectral evidence of deletions that varied between strains. This result indicates that larger deletions can be rapidly detected by observance of spectral signal alone. However, algorithms with a stringency of filtering for run length of 2 or greater will miss some deletions, and thus type II errors (false negatives) are possible. In summary, rapid filtering of data for run lengths of 2 or greater will catch most deletions, but there is a risk that false negative results will occur and that the smallest deletions will be missed using filtering approaches that attempt to avoid confirmatory sequencing.

### Description of open reading frames of PT13a *S*. Enteritidis that were disrupted

Both PT13a strains had multiple SNPs that disrupted open reading frames (ORFs) (Table 3). Using the incidence of disrupted ORFs as a measure of genetic distance from PT4 22079, PT13a 21046 is more closely related to the PT4 reference strain, because only 3 ORFs were disrupted in contrast to 12 for PT13a 21027. One mutation fused two genes, which means that a total of 16 genes, 3 for PT13a 21046 and 13 for PT13a 21027, have disrupted ORFs.

**Table 3:** SNPs of *Salmonella *Enteritidis that disrupt open-reading-frames (ORFs) and comparison of translated genes to NCBI reference sequences of *Salmonella enterica*

*Salmonella *Enteritidis gene with SNP	Type of SNP as referenced to *S*. Enteritidis PT4	Location of SNP in *S*. Enteritidis PT4 genome	*Salmonella* Enteritidis^a^			Other *Salmonella enterica *subsp. I serotypes^b,c^																	Outlier microbes		
					
						*O-antigen serotype*																			
			D1	D1	D1	D1	D1	D1	D1	A	D1	B	B	B	B	B	B	C1	C1	C2	C2	C3	na	na	na
					
			PT 13a 21046	PT13a 21027	PT4 22079 (same as reference)	Gallinarum	Dublin	Typhi	Paratyphi A	Javiana	Typhimurium LT2	Typhimurium DT104	**Typhimurium 4,**[5]**,12:i:-**	Saintpaul	Schwarzengrund	Heidelberg	Agona	Choleraesuis	Infantis	Hadar	Newport	Kentucky	*Salmonella enterica *subsp. arizonae	*Salmonella bongori*	E. coli 0157:H7 Sakai
			
*A) Polymorphisms that distinguish PT13a 21046 from PT13a 21027 and *S. Enteritidis *PT4*																									
fhuA (SEN0196)	substitution: ORF termination	226922	-	+	+	+ (78)	+ (78)	- (23)	- (23)	+ (78)	+ (78)	+ (78)	+ (78)	+ (78)	+ (78)	+ (99)	+ (100)	+ (78)	+ (78)	+ (78)	+ (78)	+ (99)	+ (97)	+ (99)	+ (93)
mocR (SEN3898)	deletion: 92 bp	4189388-4189479	-	+	+	+ (100)	+ (99)	+ (98)	+ (98)	nd	- nd	- nd	- nd	- nd	- nd	- nd	- nd	- nd	- nd	- nd	- nd	- nd	- nd	- nd	- nd
DGC (SEN4316)	deletion: 80 bp of 5' end + 135 bp upstream	4642252-4642331	-	+	+	+ (99)	+ (99)	+ (99)	+ (99)	+ (99)	+ (99)	+ (99)	+ (99)	+ (99)	+ (99)	+ (99)	+ (99)	+ (99)	+ (99)	+ (99)	+ (99)	+ (99)	+ (90)	+ (90)	- nd
*B) Polymorphisms that distinguish PT13a 21027 from PT13a 21046 and *S. Enteritidis *PT4*																									
foxA (SEN0347)	deletion: 1 bp	393529	+	-	+	- (99)	+ (99)	+ (98)	+ (99)	+ (99)	+ (99)	+ (99)	+ (99)	+ (99)	+ (99)	+ (99)	+ (99)	+ (99)	+ (99)	+ (98)	+ (99)	+ (99)	- nd	- nd	- nd
putP (SEN0989)	substitution: ORF termination	1095448	+	-	+	+ (99)	+ (100)	- nd	+ (100)	+ (100)	+ (99)	+ (99)	+ (99)	+ (99)	+ (99)	+ (100)	- nd	- (44)	+ (100)	+ (99)	+ (100)	- (nd)	+ (99)	- nd	- nd
kdgM (SEN0992)	deletion: 1 bp	1099079	+	-	+	+ (99)	+ (99)	+ (97)	+ (99)	+ (98)	+ (99)	+ (99)	+ (99)	+ (99)	+ (98)	+ (99)	- nd	+ (99)	+ (99)	+ (99)	+ (99)	- nd	+ (96)	- nd	- nd
ydcZ (SEN1464)	deletion: 6 bp	1554930	+	-	+	+ (100)	+ (99)	+ (99)	+ (98)	+ (99)	+ (99)	+ (99)	+ (99)	+ (100)	+ (100)	+ (98)	+ (99)	+ (99)	+ (98)	+ (99)	+ (99)	+ (99)	+ (95)	- nd	- nd
ydjN (SEN1723)	substitution: ORF termination	1828544	+	-	+	+ (99)	+ (99)	+ (99)	+ (99)	+ (99)	+ (100)	+ (100)	+ (100)	+ (100)	+ (99)	+ (100)	+ (99)	+ (99)	+ (100)	+ (100)	+ (99)	+ (100)	+ (99)	+ (99)	+ (96)
ytcJ-like (SEN1576)	substitution: ORF termination	1681327	+	-	+	+ (99)	+ (99)	+ (99)	+ (98)	+ (98)	+ (99)	+ (99)	na	+ (98)	+ (98)	+ (99)	+ (99)	+ (98)	+ (99)	+ (99)	+ (99)	+ (99)	- nd	+ (96)	- nd
pgk (SEN2751)	deletion: 12 bp in-frame	2939833-2939844	+	-	+	+ (99)	+ (97)	+ (98)	+ (97)	+ (99)	+ (98)	+ (98)	+ (98)	+ (98)	+ (99)	+ (98)	+ (97)	- (80)	+ (97)	+ (98)	+ (98)	+ (97)	+ (99)	+ (85)	- nd
cysN (SEN2773)	substitution: ORF termination	2957802	+	-	+	+ (99)	+ (99)	+ (99)	+ (99)	+ (99)	+ (99)	+ (99)	+ (99)	+ (99)	+ (99)	+ (99)	+ (99)	+ (99)	+ (99)	+ (99)	+ (99)	+ (99)	+ (99)	+ (92)	+ (87)
dsdA (SEN3619)	deletion: 10 bp	3880104-3880113	+	-	+	+ (100)	+ (99)	+ (99)	+ (99)	+ (99)	+ (99)	+ (99)	+ (99)	+ (99)	+ (99)	+ (100)	+ (99)	+ (99)	+ (99)	+ (99)	+ (100)	+ (99)	+ (97)	- nd	- nd
lrgB (SEN4042)	deletion: 12 bp in-frame	4368126-4368137	+	-	+	+ (100)	+ (99)	+ (99)	+	+ (100)	+ (100)	+ (100)	+ (100)	+ (100)	+ (100)	+ (100)	+ (100)	+ (100)	+ (100)	+ (100)	+ (100)	+ (100)	+ (99)	+ (97)	- nd
yjfK (SEN4139)	shared deletion: fusion of yjfK and yjfL; ribosomal binding site of yjfL removed	4472579-4472589	+	-	+	+ (99)	+ (99)	+ (99)	+ (99)	+ (99)	+ (99)	+ (100)	+ (100)	+ (100)	+ (99)	+ (100)	+ (99)	+ (99)	+ (100)	+ (100)	+ (100)	+ (99)	- nd	- (89)	- nd
yjfL (SEN4140)			+	-	+	- nd	+ (100)	+ (99)	+ (100)	+ (99)	+ (100)	+ (100)	+ (99)	+ (100)	+ (99)	+ (100)	+ (99)	+ (99)	+ (100)	+ (100)	+ (100)	+ (99)	- (nd)	+ (98)	- (nd)
sefD (SEN4250)	deletion: single bp	4574262	+	-	+	- (99)	+ (100)	- (99)	- (99)	- nd	- nd	- nd	- nd	- nd	- nd	- nd	- nd	- nd	- nd	- nd	- nd	- nd	- nd	- nd	- nd
^a ^Abbreviations and symbols: na, possible error in contig assembly or database or otherwise not applicable; nd, no similar gene detected; nucleotide sequence similarity rather than amino acid sequence similarity is included for genes that are pseudogenes, which are recorded by a "-" even if percent nucleotide similarity is high.																									
^b ^Numbers in parentheses indicate % similarty between translated amino acid sequences of S. Enteritidis in comparison to indicated serotype, unless a pseudogene is present and reported as nucleotide similarity.																									
^c ^*Strains and databases referenced are:*																									
Serotype designation						Refseq:	Genbank accession																		
Salmonella enterica subsp. arizonae serovar 62:z4,z23:--, complete genome						NC_010067	CP000880																		
Salmonella enterica subsp. enterica serovar 4,[5],12:i:- str. CVM23701						NZ_ABAO00000000	ABAO00000000																		
Salmonella enterica subsp. enterica serovar Agona str. SL483						NZ_ABEK00000000	ABEK00000000																		
Salmonella enterica subsp. enterica serovar Choleraesuis str. SC-B67						NC_006905	AE017220																		
Salmonella enterica subsp. enterica serovar Dublin str. CT_02021853						NZ_ABAP00000000	ABAP00000000																		
Salmonella enterica subsp. enterica serovar Heidelberg str. SL476						NC_011083	CP001120																		
Salmonella enterica subsp. enterica serovar Heidelberg str. SL486						NZ_ABEL00000000	ABEL00000000																		
Salmonella enterica subsp. enterica serovar Javiana str. GA_MM04042433						NZ_ABEH00000000	ABEH00000000																		
Salmonella enterica subsp. enterica serovar Kentucky str. CDC 191						NZ_ABEI00000000	ABEI00000000																		
Salmonella enterica subsp. enterica serovar Kentucky str. CVM29188						NZ_ABAK00000000	ABAK00000000																		
Salmonella enterica subsp. enterica serovar Newport str. SL254						NC_011080	CP000604																		
Salmonella enterica subsp. enterica serovar Newport str. SL317						NZ_ABEW00000000	ABEW00000000																		
Salmonella enterica subsp. enterica serovar Paratyphi A str. ATCC 9150						NC_006511	CP000026																		
Salmonella enterica subsp. enterica serovar Saintpaul str. SARA23						NZ_ABAM00000000	ABAM00000000																		
Salmonella enterica subsp. enterica serovar Saintpaul str. SARA29						NZ_ABAN00000000	ABAN00000000																		
Salmonella enterica subsp. enterica serovar Schwarzengrund str. CVM19633						NC_011094	CP001127																		
Salmonella enterica subsp. enterica serovar Schwarzengrund str. SL480						NZ_ABEJ00000000	ABEJ00000000																		
Salmonella enterica subsp. enterica serovar Typhi Ty2						NC_004631	AE014613																		
Salmonella enterica subsp. enterica serovar Typhi str. CT18						NC_003198	AL513382																		
Salmonella typhimurium LT2						NC_003197	AE006468																		
E. coli 0157:H7 Sakai						NC_002695	BA000007																		
*Sanger Institute databases are available at *http://www.sanger.ac.uk/Projects/Salmonella/.																									
Salmonella bongori 12419 ATCC 43975																									
Salmonella enterica Enteritidis PT4 NCTC 13349																									
Salmonella enterica Gallinarum 287/91 NCTC 13346																									

### Assay of *S*. Enteritidis SNPs that differentiate within phage type across *Salmonella enteric*

Table 3 summarizes results from BLAST searches of disrupted ORFs across publicly available *Salmonella enterica *databases. The database of serotype genomes is in general heavily skewed towards mutation in *mocR *and *sefD; *in addition, *fhuA *has two alternative sequences that varies among serotypes. Each *Salmonella *serotype appears to have its own unique combination of ORFs that vary in completeness. For example, *S*. Typhi lacks *fhuA *and *putP*, whereas *S*. Kentucky lacks *mocR, putP *and *kdgM. E. coli *lacks 13 of the 16 genes listed in Table 3; thus, this subset of genes may be more likely to be linked to the biology of *Salmonella enterica *than it is to the biology of either *E. coli *and perhaps *Shigella. Salmonella bongori *lacks 8 of the 16 genes, which suggests that the subset of genes is more a marker of *Salmonella *causing illness than it is to environmental adaptation. Finally, *Salmonella enterica *subsp. Arizonae did not have complete ORFs for 6 of the genes in the subset, which suggests that this pathogen more associated with colonization of reptiles and amphibians has also evolved differently in regards to genes impacted by these SNPs.

## Discussion

### Importance of starting analyses with characterized strains

The 3 strains chosen for this comparative approach varied in a number of phenotypic assays, which was used as *prima facie *evidence that the strains must vary in genetic content even in the absence of specific knowledge of the differences. Rejection of the concept that there was an identifiable difference in genomic content would have required rejecting basic tenants of evolutionary theory. However, this research does not claim that any one genetic difference causes any one phenotype in the absence of further research to establish biological role. It is likely that phenotypes observed with various assays require a combination of genetic events. Supplementary material in Additional File 1 provides a searchable database for investigators interested in acquiring specific information on any one gene, which includes information on KEGG pathways and gene function.

### Linkage of genotype to phenotype requires stringent application of comparative genome approaches and phenotypic analyses

Detection of SNPs that may be causally associated with phenotype of pathogenic bacteria is an inherently problematic undertaking, because a change of only 1 bp out of millions has the potential to alter the biology of the bacterium [24]. If the genetic distance between two organisms is too much, the ability to link a phenotype to a genotype is complicated by the numbers of polymorphisms that will be found. In other words, the challenge of the bacterial genome is its sheer capacity to rapidly accumulate polymorphisms, either by random genetic drift, selection of specific genetic capabilities, or by lateral transfer of sets of polymorphisms through events such as homologous recombination or acquisition of genes from extrachromosal DNA [12]. By triangulating 2 genomes of strains within a single phage type to that of a genome from a different phage type, genetic noise was reduced and a discrete number of non-synonymous polymorphisms were found that differentiated the two PT13a strains. Selection of strains was key to the success of the approach and multiple phenotypic assays were used to select stable strains with variant phenotypes [12].

It must be noted that there are more subpopulations to be characterized. Also, only a few of the SNPs listed here are likely to have epidemiological significance and/or a causal link to virulence attributes. Further analysis that compares mutant to complemented mutant and parent strain is required to identify markers of highest value for causing anyone phenotypic attribute. ORF disrupting mutations are emphasized as being especially important in future research, because they introduce an obvious change in genome content. Obtaining information about which SNPs occur frequently within the environment of poultry and that can be linked to egg contaminating phenotypes is also important, because these can be genetic markers that may facilitate epidemiological investigations. Research is in progress to evaluate if SNPs described here are commonly found in clinical and environmental strains and to determine their biological impact. It is expected that many SNPs will be inconsequential.

The ability to find a discrete set of SNPs was dependent on extensive research completed prior to initiating genomic analyses. For example, biofilm production was selected as an indicator of potential virulence, because it is often cited as contributing to the ability of bacterial pathogens to colonize, survive and persist in hosts [25-28]. Phenotype microarray was a second assay that more specifically and quantitatively defined how strains varied in regards to metabolic properties [11]. Finally, the use of a highly relevant animal model, namely the hen infection model, increased the ability to keep analyses focused on traits impacting egg contamination. In contrast, mouse models are inherently limited in their ability to assess genetic factors impacting egg contamination, because mice lack an oviduct and the reproductive cycle of a hen. Other approaches to studying phenotype include tissue cell assays, injecting bacterial cells into egg contents and coating the shell of eggs with cells [29]. While these methods help identify sets of polymorphisms and biological properties specific to a narrow niche, they are unlikely to identify the broader set of polymorphisms required for *S*. Enteritidis to complete an entire infection pathway that results in egg contamination. It thus appears important for a number of phenotypic assays to be used to characterize strains [18]. A change in 1 to 2% of whole gene content, or about 1 gene of every 100, is a point where niche specialization of *S*. Enteritidis impacting egg contamination is apparent.

### Application of high throughput technology is not complete without a discussion of limits

It is important to assess how likely automated methods of genome analysis will generate false positive and false negative results, because high throughput methods can rapidly propagate misinformation. High-density tiling mutational mapping alone was subject to Type I (false positive) error, whereas comparative genome re-sequencing (CGS) was subject to Type II (false negative) error. Pyrosequencing was subject to Type II error for ribosomal gene base pair substitutions and deletions, but was otherwise accurate for calling base pair substitutions. Mutational mapping was overall the more cost-effective and accurate method for finding true polymorphisms that differentiated PT13a strains 21027 and 21046 as confirmed by sequencing f/r strains PT13a 21027, PT13a 21046 and PT4 22079. The cost of sequencing was greatly reduced by incorporating an approach that reduced genetic noise, namely triangulation of 3 genomes within the same serotype for virtual subtractive hybridization. Combining mutational mapping with pyrosequencing may eventually be the most cost effective and time efficient approach for SNP analysis of bacteria that have an exceptionally clonal population structure. Finding that pyrosequencing was excellent for detection of SNPs that involve basepair substitutions suggests that it may be a preferred method for epidemiological investigations, because it is amenable to being automated. However, if certain SNPs occur repeatedly within different subpopulations, then targeted sequencing may be more cost effective. For example, if epidemiological tracebacks could be accomplished using as few as 10 SNPs to track an outbreak strain, a baseline cost would be approximately $100 per strain ($10/SNP). It is not yet possible to complete whole genome sequencing for such a low cost, although it is required for the initial identification of SNPs useful for building analytical platforms. Application of these methods requires acknowledgement of method limitations and a strategy for overcoming deficiencies and skew in order to produce the highest quality databases.

### Mutational analysis, development of 2^nd ^generation vaccines and future research objectives

The relative contribution of each non-synonymous SNP to the pathway that results in egg contamination will be better understood as defined mutants within a similar genomic background are characterized. Knowledge of how combinations of genes aid organ colonization and growth will help to choose strains that are suitable for development of vaccines. The possibility that there is a vaccination strategy that prevents multiple serotypes from colonizing and growing in internal organs should be explored, because serotypes other than *S*. Enteritidis could evolve the ability to contaminate eggs. These results support the conclusion that *S*. Enteritidis has multiple evolutionary trajectories, involving multiple polymorphisms throughout the genome, which impart combinatorial complexity [12]. Finding which SNPs impact the ability of *S*. Enteritidis to complete the infection pathway to the egg and ultimately to the consumer is an objective of future research.

## Conclusions

The conundrum of how *Salmonella enterica *serovar Enteritidis generates an exceptional degree of strain heterogeneity while exhibiting a highly clonal population structure has been solved for the most part. *S*. Enteritidis appears to be undergoing evolution primarily at the level of the single nucleotide polymorphism. Even bacteriophage lineages share most chromosomal information outside of regions of lysogeny. This result indicates that exceptionally stringent methods for analysis of genetic variation are required for characterization of strains. This research supports concepts of evolution that have been previously published [12]. Linking variant genotypes to distinctive phenotypes remains a focal area of research intended to characterize evolutionary events that enable *S*. Enteritidis to contaminate the internal contents of eggs produced by otherwise healthy hens.

## Methods

### Strains for analysis

The PT4 22079 was an environmental isolate obtained from water downstream from a flock and traced back to a major outbreak of egg contamination that was historically associated with introduction of PT4 to the United States [14]. The two PT13a strains were from the spleen of a rodent caught in a hen house located in the Northeast of the United States, which is a region that had been heavily impacted by the 1980s outbreak of *S*. Enteritidis. PT13a 21046 was shown to contaminate eggs, but it did not form biofilm; conversely, PT13a 21027 formed biofilm, but it did not contaminate eggs [12]. PT4 22079 contaminated eggs and formed biofilm, but its biofilm was weak in comparison to PT13a 21027 [11] (Figure 1). The two PT13a strains are clonally related, because no differences in overall gene content could be detected by microarray analysis [11,12]. The summation of phenotypes for strains PT13a 21046, PT13a 21027 and PT4 22079 based upon biofilm (BF) and egg contamination (EC) is BF- EC+, BF+ EC-, and BF+ EC+, respectively. Gene nomenclature for *S*. Enteritidis is SEN plus a 4 digit number that indicates relative position within in the genome. SEN0001 is *thrA *and the last gene is SEN4356A, which overlaps *thrA*. Ribosomal genes are accessioned separately, from SEN_r001 to SEN_r022 for ribosomal RNA and SEN_t001 to SEN_t084 for transfer RNA [8].

### Differentiation of strains by biofilm phenotype

The three strains varied in biofilm production, which facilitated confirming the presence of different genomic variants. Colony morphologies for the three strains were previously published, but are shown again here to emphasize a type of phenotypic variation commonly encountered in cultures of *S*. Enteritidis that cannot be detected without following culturing methodology (Figure 1) [12]. A review of the literature suggests that our laboratory uses a technique not in general use [17]; thus, other laboratories may not be aware that they have the same phenotypes within a single culture. The biofilm-forming characteristics of each strain used herein were confirmed as follows. Colonies from primary plates were transferred to brilliant green (BG) agar (Acumedia-Neogen, Lansing, MI, USA) and inoculated at 10 different spots per plate so that colonies were well spaced. Spacing is required to allow colonies to grow large enough to form a well-developed biofilm. Plates were incubated for 16 hr at 37°C and then transferred to ambient temperature (24 to 27°C). Colonies were scored for morphology at 24 hr intervals and given a final classification following a total of 120 hr incubation as having: i) strong biofilm formation, indicating colonies formed a well-developed organic matrix that covered the entire colony beginning within 48 hr of incubation at ambient temperature, ii) smooth, indicating colonies formed no biofilm, or iii) weak, indicating biofilm formation was not apparent until after 48 hr at room temperature and it became more organized as incubation progressed. A strain that was classified as weak never formed a biofilm resembling one that was classified as strong. Colony images were recorded using the Molecular Imager ChemiDox XRS (Biorad) with epi white light and auto-capture. Whole colony images were digitally edited to increase contrast for purposes of publication and have been previously published [17].

### Pulsed field gel electrophoresis (PFGE)

The PFGE patterns for PT13a 21027, PT13a 21046 and PT4 22079 were determined using standardized methods conducted by personnel trained in the method developed by the Centers for Disease Control as part of PulseNet http://www.cdc.gov/pulsenet/references.htm[30-32]. Briefly, bacterial genomic DNA plugs were digested using the restriction enzyme, *Xba*I (Promega, Madison, WI, USA). Digested DNA was separated using the CHEF-DRII PFGE system as per the manufacturer's instructions (Bio-Rad, Hercules, CA, USA). Electrophoresis was carried out for 19 h at 6 V, using 2.2 L of the buffer× Tris/borate/EDTA0.5 (TBE) at a temperature of 14°C, and an initial pulse time of 2.16 s followed by a final switch time of 63.8 s. BioNumerics software (Applied Maths Scientific Software Development, Belgium) was used to normalize the band patterns based on the molecular weight standards included on each gel.

### DNA isolation

Single colonies of *S*. Enteritidis strains were grown in 10 ml of Brain Heart Infusion broth (BHI) (Difco BD, Franklin Lakes, NJ) at 37°C for 16 hr. Bacterial cells were pelleted in a Sorvall RC5B Plus centrifuge at 5000 × *g *for 15 min in a Sorvall Super-lite SLA 600 TC rotor. For mutational mapping and associated re-sequencing services (Nimblegen, Inc.), total DNA was extracted using a Qiagen Genomic-tip 500/G kit following the protocol designated for bacteria. Precipitated DNA was dissolved in 150 ml of Tris-EDTA buffer (10 mM Tris-HCl, 1 mM EDTA [pH 8]) and stored at -20°C. For confirmatory sequencing from PCR-amplified product, total DNA was extracted using a Qiagen Genomic-tip 100/G kit following the protocol designated for bacteria (Qiagen, Valencia, Calif.). Precipitated DNA was dissolved in 200 ml of Tris-EDTA buffer (10 mM Tris-HCl, 1 mM EDTA [pH 8]) and stored at -20°C. Spectrophotometric readings were performed to ensure an OD260/280 ratio greater than 1.7 and a genomic DNA concentration of 1 μg/μl as required for CGS.

### Whole genome analysis and confirmatory sequencing of putative polymorphisms

*Salmonella enterica *has seven ribosomal regions that are highly similar and which can complicate assembly and annotation of libraries. For this reason, a high density tiling method was selected for first analysis over 454 sequencing, because it was less likely to produce assembly artifact due to its progressive nature. Whole genome analysis was divided into two phases as described by the provider of service (Nimblegen, Inc.; http://www.nimblegen.com/products/cgr/index.html). Briefly, the first phase is called mutational mapping. A set of 30-mer probes is computer generated from a reference sequence. For these analyses, the database of the *S*. Enteritidis PT4 reference genome from the Pathogen Sequencing Group at the Sanger Institute was used for generation of primers [8] (GenBank AM933172) and DNA for the experimental protocol was from PT4 22079. The primer set is used to densely tile the test genome that has been labeled with a fluorescent signal. The probes overlap every 7 bases. For a genome that is approximately 5 million base pairs, approximately 1.42 million primers are required for tiling hybridization, with the service provider stating that chip capacity is about 1.3 million base pairs of genome and 385,000 probes. Our experiments required 4 chips to process the entire genome of *S*. Enteritidis, which is approximately 4.86 million base pairs. Genomic regions identified by mutational mapping are then fed into the second phase of analysis, which is referred to as targeted resequencing. Any location of the test DNA that has a suspected SNP is resequenced in the presence of all 4 nucleotides to see which one allows sequencing to progress. Since the nucleotides are labeled, it is possible to tell which nucleotide is incorporated and thus identity is established.

Although the two phases are meant to progress seamlessly, we did them separately to evaluate datasets at each stage for sensitivity and stringency in regards to detection of polymorphisms. Raw data were provided in file format that were accessed using proprietary software (SignalMap Versions 1.8 or 1.9 by Nimblegen, Inc.). To facilitate analysis, numerical signal data available as tab-format text files and readable with Notepad (Microsoft) were opened and transferred to multiple spreadsheets in 400 kb sections. 3 SNPs in the genes *rrlC*, *rrlA*, and *cyaA *had previously been found by use of a modified ribotyping approach [33]. All three of these known SNPs, which served as internal controls, were detected by Phase I mutational mapping.

Phase II of CGS is an array-based re-sequencing process that identifies which single base pair is substituted for another and at what position the substitution or other change occurs in the reference genome. The known polymorphisms in *rrlC, rrlA *and *cyaA *were again used as internal controls for assessing specificity and sensitivity of mutational mapping done in Phase I and confirmatory resequencing done in Phase II. All signals for the control SNPs generated during Phase II fell into the category "non-called ROI". This meant that Phase II re-sequencing technology resulted in many false negatives. Therefore, SNPs that fell into the "non-called ROI" category were analyzed by confirmatory PCR-based sequencing in forward and reverse directions.

Pairs of primers used to amplify DNA amplicons for confirmatory sequencing of putative polymorphisms were generated from the *S*. Enteritidis PT4 whole genome sequence made publicly available by the Pathogen Sequencing Unit of the Sanger Institute (EMBL accession no.: AM933172) [8]. Forward and reverse primers used for confirmatory sequencing are available as catalogued information at the National Center for Biotechnology Information (NCBI) and identified within dbSNP by Assay ID.

The cycling conditions for an Applied Biosystems 2400 Gene Amp PCR system were determined individually for each primer pair and in general included denaturation at 95°C for 1 min, then 30 cycles of 95°C for 30 s, 60°C to 70°C for 30 s, and 72°C for 2 to 3 min. Each reaction contained 400 nM of each primer, 200 μM ACGT deoxynucleotide triphosphates (dNTPs), 1.5 mM Mg^++^, 2.5 U Taq enzyme (Fisher, Pittsburg, Pa.), and 1μl template DNA. Single amplicon products were confirmed by gel electrophoresis. PCR products were purified using a Qiagen QIAquick PCR Purification kit and submitted for sequencing to the Eastern Regional Research Center (Wyndmoor, PA, USA). PCR amplicons were sequenced using an Applied Biosystems BigDye Terminator 1.1 reaction mix on an Applied Biosystems 3730 DNA Analyzer.

Pyrosequencing (SeqWright) was performed according to the manufacturer's instructions following methods developed for the sequencing of bacteria with a genome of approximately 5 MB [34]. At least 10 ug of DNA was submitted for each strain. DNA quality was confirmed to have an OD260/280 ratio > 1.8 and a minimum concentration of 50 ng/uL in TE as measured by spectrophotometer (NanoDrop). Briefly, sample was fragmented to between 300-500 bp and ends were repaired. Adaptor ligation was used to tag fragments with a 5'-biotin tag. Library beads were emulsified with amplification reagents in an oil water mix. After amplification, library beads were layered onto a PicoTiterPlate™. Single-stranded PCR products were sequenced using a GS FLX XLR70 Titanium platform (454 Life Sciences) and product from all 3 strains was run on one full plate. Data assembly was done with commercially available software (Newbler^™ ^Assembler).

### Sequence alignment, similarities, and phylogenetic analysis

Nucleotide and amino acid sequence alignment was done by the Clustal W method using software from DNASTAR. Default parameter settings were used. Nucleotide polymorphisms were located within the PT4 reference genome using the EditSeq and SeqMan programs. The nucleotide location of interest ± 150 bp of flanking DNA was used to conduct BLAST searches against the *S*. Typhimurium LT2 genome available at NCBI (GenBank: AE006468). If no match with similarity greater than 90% was found between the two serotypes, the BLAST search was first extended to other *Salmonella enterica *serotypes with available whole genome databases, then to all available *Salmonella *databases and finally to all proteobacteria in the gamma subdivision. Additional BLAST searches were conducted on other databases available at the Sanger Institute.

Once it was determined that a polymorphism was within a gene, all available sequences for that gene were downloaded to EditSeq files from completed *Salmonella *genomes available as public databases from NCBI http://www.ncbi.nlm.nih.gov/sutils/genom_table.cgi and The Sanger Institute http://www.sanger.ac.uk/cgi-bin/blast/submitblast/Salmonella. Gene sequences were translated to amino acid sequences and aligned with DNAStar MegAlign software. Results are shown in Table 2. Attempts were made to annotate genes and to identify genes flanking non-coding regions based upon available annotation from both *S*. Enteritidis and *S*. Typhimurium. Linkages to proteins and inclusion of some information about conservation of amino acid change, gene class and gene function is included in supplemental information (Additional File 1).

### Construction of a SNP interval map

Any gene with a SNP that differentiated the two PT13a strains was annotated by gene accession number according to its location in the *S*. Enteritidis PT4 reference database and according to similarity to genes in *S*. Typhimurium LT2 [35]. SNPs in intergenic regions were included in the interval map by annotating with the 3' gene flanking the polymorphism with the extension ".5" after annotation. The number of genes between *S*. Enteritidis PT13a genes containing SNPs was determined using a Microsoft Excel automated sequential function to calculate the difference between gene accession numbers for the two serotypes. To do this, the gene annotations were numbered from least to greatest for *S*. Enteritidis PT4, and then these genes were aligned with the similar gene from *S*. Typhimurium LT2. It is important to note that the genes were initially selected as similar pairs by BLAST search; however, the order within respective genomes can be different due to inversions [7,21].

## List of Abbreviations

aa: amino acid,; ap: acidic polar; BG: brilliant green; BHI: brain heart infusion; bp: basic polar; CGS: comparative genome sequencing; dNTPs: deoxynucleotide triphosphates; EMBL: The European Molecular Biology Laboratory; HMM LPS: high-molecular-mass LPS; LPS: lipopolysaccharide; NCBI: National Center for Biotechnology Information; nn: neutral non-polar; np: neutral polar; PCR: polymerase chain reaction; PFGE: pulsed field gel electrophoresis; PT: phage type; *S*. Enteritidis: Salmonella enterica serovar Enteritidis; *S*. Typhimurium: Salmonella enterica serovar Typhimurium; SNP: single nucleotide polymorphism

## Disclosure of Competing interests

The authors declare that they have no competing interests.

## Authors' contributions

JG conceived of, designed and implemented the approach used to conduct whole genome sequencing in a manner to minimize genetic noise. JG previously conducted animal and phenotypic experiments with technical staff. JG developed the biofilm assay. JG collated and managed all data, reviewed methods for accuracy, chose providers of services, and drafted the manuscript. JG developed strategies for animal and phenotype assays that led to selection of strains. CM designed primers, carried out confirmatory sequencing and performed phenotype microarray assays. CM participated in reviewing drafts of the manuscript and to the management of data. CM managed the laboratory on a daily basis, maintaining reagents and working stocks. PFC conducted PFGE analysis. RKG participated in related animal experimentation. All authors read and approved the final manuscript.

## Supplementary Material

Additional file 1 **Single nucleotide polymorphisms (SNPs) of *Salmonella enterica *subsp**. I serovar Enteritidis PT13a that differentiate subpopulations within variant pathotypes. This file is a searchable spreadsheet that lists each confirmed SNP according to its location in reference strain *Salmonella enterica subsp. enterica serovar str. P125109*NC_011294 (GenBank AM933172). An investigator can type in a gene name or label under the "find" function under "Edit" in Word Excel to see if the gene they are interested in contains a SNP. Column headings indicate specific information about each SNP, including if it is synonymous or non-synonymous. This is the file that will be updated in the event errors or additions are reported or discovered within the database. Click here for file

Additional file 2 **Interval map aligning SNPs of *Salmonella *Enteritidis PT13a (SEN) to the genome of *Salmonella *Typhimurium LT2 (STM)**. This file was generated to search for gaps between genes that might indicate an insertion or deletion that was missed by the mutational mapping approach. Primers were generated only from the reference sequence. If the test sequence has additional DNA not present in the reference sequence, it could go unnoticed. Regions offset by 5 genes or greater suggest different gene contents. See text for further information. Click here for file
