# Supplementary material for: Single nucleotide polymorphisms that differentiate two subpopulations of Salmonella enteritidis within phage type
Source: BMC Res Notes. 2011 Sep 26;4:369. doi: 10.1186/1756-0500-4-369 (PMC3220660; doi:10.1186/1756-0500-4-369)
Supplement: Additional file 2 — Interval map aligning SNPs of Salmonella Enteritidis PT13a (SEN) to the genome of Salmonella Typhimurium LT2 (STM). This file was generated to search for gaps between genes that might indicate an insertion or deletion that was missed by the mutational mapping approach. Primers were generated only from the reference sequence. If the test sequence has additional DNA not present in the reference sequence, it could go unnoticed. Regions offset by 5 genes or greater suggest different gene contents. See text for further information. [file 1756-0500-4-369-S2.PDF]

Additional File 2. Interval map aligning SNPs of *Salmonella* Enteritidis PT13a (SEN) to the genome of *Salmonella* Typhimurium LT2 (STM)

SEN gene or region with SNP[a,b,c]	Number of genes separating PT13a SNPS	STM gene similar to SEN gene	Number of STM genes separating PT13a SNPs	Gap in alignment of SEN and STM genes[b]	Gene name[a] (number of SNPs)
65	na	64	na	1	dapB
72	7	71	7	0	caiC
83.5	11.5	83.5	12.5	-1	STM0083-0084 (2)
112	28.5	111	27.5	1	leuC
161.5	49.5	157.5	46.5	3	yacH-acnB
170	8.5	165	7.5	1	speD
177	7	172	7	0	yadG
196	19	191	19	0	fhuA
205	9	200	9	0	stfG
208	3	203	3	0	yadQ
219	11	213	10	1	dapD
230	11	223	10	1	yaeL
261	31	259	36	-5	yafE
311	50	328	69	-19	none
347	36	364	36	0	foxA
353	6	370	6	0	prpD
354	1	371	1	0	prpE
416	62	434	63	-1	apbA
450	34	469	35	-1	rpmE2
458	8	477	8	0	acrR
475	17	494	17	0	ushA
503	28	522	28	0	allP
505	2	524	2	0	ybbY
569	64	600	76	-12	cstA
578	9	609	9	0	ahpF
594	16	625	16	0	dpiB
596.5	2.5	627.5	2.5	0	dcuC-pagP
619	22.5	650	22.5	0	uxaA
629	10	660	10	0	pseudo
648.5	19.5	684.5	24.5	0*	nagB-nagE
650.5	2	686.5	2	0	glnS-ybfM
712	61.5	766	79.5	-18	dcoA-2 (9)
721	9	776	10	-1	galE
738	17	793	17	0	bioA
751	13	805	12	1	moaD
753	2	807	2	0	ybhL
759.5	6.5	813.5	6.5	0	ybhP-ybhQ
798	45	852	45	0	yliG
814	54.5	868	54.5	0	deoR

824	26	878	26	0	potG
836.5	22.5	891.5	23.5	-1	artP-ybjP
850	26	942	64	-38	yjbZ
917	80.5	1053	161.5	-81	none
930	13	1065	12	1	ymbA
930.5	13.5	1065.5	12.5	1	ymbA-rmf
989	59	1128	63	-4	putP
992	61.5	1131	65.5	-4	kdgM
1025.5	33.5	1984.5	na	na	yedP-yodD
t021-t022	na	1943.5	na	na	cysT-glyW
1067	41.5	1938	46.5	-5	yecA
1068	1	1937	1	0	tyrP
1068.5	0.5	1936.5	0.5	0	tyrP-yecH
1081	12.5	1922	14.5	-2	motB
1093	12	1910	12	0	ftsI
1118	25	1885	25	0	edd
1120.5	2.5	1882.5	2.5	0	purT-yebG
1162	41.5	1869	13.5	28	none
1164	2	1868	1	1	none
1194	30	1843	25	-1*	none
1199	5	1838	5	0	yobF
1201	2	1836	2	0	ftsI
1204	3	1833	3	0	none
1209	5	1828	5	0	yoaE
1210	1	1827	1	0	none
1238	28	1799	28	0	emtA
1241	3	1796	3	0	treA
1250	9	1787	9	0	none
1279.5	29.5	1756.5	30.5	-1	purU-tyrT
1295.5	16	1738.5	18	-2	cls-yciI
1327.5	32	1705	33.5	-1.5	osmB
1355	27.5	1678	27	0.5	none
1386	31	na	na	na	none
1418	32	1635	43	-11	none
1446	28	1610	25	3	ydcK
1454.5	8.5	1601.5	8.5	0	sifB-ugtL (2)
1464	9.5	1591	10.5	-1	ydcZ
1480.5	16.5	1574.5	16.5	0	tetR-smvA
1484	3.5	1571	3.5	0	yddG
1509	25	1543	28	-3	none
1539	30	1512	31	-1	dcp
1545.5	6.5	1504.5	7.5	-1	rspA-ynfA
1555	9.5	1495	9.5	0	ynfI
1558	3	1491	4	-1	none
1575.5	17.5	1472.5	18.5	-1	ompN-STM1472
1576	0.5	1472	0.5	0	ytcJ-like
1590	14.5	1457	15	-0.5	rnfC
1611	21	1436	21	0	nemA

1619	8	1428	8	0	ydhC
1636	17	1409	19	-2	ssaJ
1663	27	1382	27	0	orf 408
1675	12	1370	12	0	sufB
1683	8	1361	9	-1	ydiM
1695	12	1349	12	0	pps (2)
1700	5	1344	5	0	ydiV
1723	23	1320	24	-1	ydjN
1737	14	1306	14	0	astB
1753	na	na	na	na	SeD_A2056
1799	62	1252	54	8	none
1800.5	1.5	1250.5	1.5	0	STM1251-1250
1805.5	5	1242.5	8	-3	cspH-envE
1831	25.5	1218	24.5	1	lolD
1883	52	1165	53	-1	grxB
1885.5	2.5	1162.5	2.5	0	pyrC-dinI
1905.5	20	1143.5	19	1	csgB-csgD
1906	0.5	1142	1.5	-1	csgD
1970	na	na	na	na	SeD-A2305
1995	na	na	na	na	SeD_A2331
2050	na	2052	na	na	pduQ
2060.5	10.5	2062.5	10.5	0	dacD-phsC
2063	2.5	2065	2.5	0	phsA
2065	2	2066	1	1	sopA
2075	10	2076	10	0	hisA
2085	na	na	na	na	rfbX (SeD_A2427)
2093	18	2097	21	-3	rfbB (2)
2108	15	2112	15	0	wcaD
2111	3	2115	15	-12	wcaA
2129	18	2134	15	3	none
2150	21	2156	15	6	none
2185	35	2192	36	-1	yeiB
2197	12	2204	12	0	fruA
2344	147	2362	158	-11	purF
2396	52	2410	48	4	yfeA
2427	31	2446	36	-5	none
2438	11	2458	12	-1	eutB
2444	6	2464	6	0	eutN
2473.5	29.5	2492.5	28.5	1	cdaR-perM
2494	20.5	2514	21.5	-1	ratB
2510	16	2530	16	0	none
2516	6	2536	6	0	pepB
2527	11	2547	11	0	none
2531	4	2551	4	0	nicO
2570	39	2644	93	-54	srmB
r005	na	2657	na	na	rrlG (2)
2591.5	10.5	2670.5	16.5	-6	aroF-yfiR (2)
2609	17.5	2689	18.5	-1	bapA (2)

2638	29	2794	105	-76	ygaE
2662	24	2817	23	1	luxS
2680	18	2839	22	-4	ygaA
2721	41	2879	40	1	sicP
2751	30	2912	33	-3	pgk
2773	22	2934	22	0	cysN
2819	46	2974	40	6	fucA
2834	15	2992	18	-3	argA
2843	9	3001	9	0	thyA
2853.5	10.5	3010.5	9.5	1	aas-galR
2867	13.5	3024	13.5	0	rcnA
2874	7	3030	6	1	none
2875	1	3031	1	0	none
2928	53	3084	53	0	fadR
2942	14	3099	15	-1	yggR
2949	7	3106	7	0	ansB (2)
2986	37	3143	37	0	hybG
3000	14	3157	14	0	yghA
3011	11	3168	11	0	ygiR
3017	6	3174	6	0	parC
3082	65	3241	67	-2	tdcE
3148	66	3315	74	-8	yrbH
3163	15	3330	15	0	gltB
3191	28	3358	28	0	none
3213	22	3379	21	1	accB
3223	10	3389	10	0	envR
3225	2	3391	2	0	acrF
3230	5	3402	11	0*	yrdC
3302	72	3475	73	-1	nirD
3353	51	3528	53	-2	pseudo
3355	2	3531	3	-1	none
3360	5	3536	5	0	glgC
3361	1	3537	1	0	glgX
3367	6	3544	7	-1	yhhW
3387	20	3564	20	0	livK
3427	40	3604	40	0	none
3438	11	3615	11	0	yhjK
3442	4	3619	4	0	bcsA
3487	45	3665	46	-1	avtA
3497	10	3675	10	0	sgbH (2)
3533	36	3711	36	0	rfaF
3537	4	3715	4	0	rfaZ
3538	1	3716	1	0	rfaY
3541	3	3719	3	0	rfaB
3587	46	3765	46	0	yicL
3608	21	3792	27	-6	none
3619	11	3802	10	1	dsdA
3676.5	57.5	3862.5	60.5	-3	glmU-STM3683
3703.5	27	3888.5	26	1	yieP-rrsC

r012	na	3891	na	na	rrlC (4)
3721.5	18	3915.5	27	-9	trxA-rho
3730	8.5	3925	9.5	-1	wecE
3740	10	3939	14	-4	cyaA (2)
3749	9	3952	13	-4	corA
r014-t068	na	3988.5	na	na	rrsA-ileT (3)
t068	na	3989	na	na	ileT
r015	na	3991	na	na	rrlA (3)
3843	94	4052	100	-6	pseudo
3857	14	4067	15	-1	none
3864	7	4074	7	0	ego
3898	na	na	na	na	mocR subfamily (SeD_A4510)
3925.5	61.5	4131.5	57.5	4	murI-SEN_r017
r021	na	4179	na	na	rrlE (2)
3965	39.5	4183	51.5	-12	aceB
3986	21	4220	37	-16	lysC
4023	37	4254	34	3	uvrA
4030	7	4261	7	0	siiE
4039	9	4269	8	1	yjcE
4042	3	4272	3	0	lrgB
4071	29	4299	27	2	melB
4086	15	4315	16	-1	hilD
4087	1	4316	1	0	none
4090	3	4319	3	0	phoN
4139	49	4373	54	-1	yjfK*
4140	1	4374	1	0	yjfL*
4177.5	37.5	4408.5	34.5	3	msrA-ytfM
4187	9.5	4438	29.5	-20	pmbA
4204	na	na	na	na	treB (SeD-A4835)
4232	54.5	4479	70.5	-16	yjgP
4239	52	4486	48	4	yjgB
4250	na	na	na	na	sefD
4256	na	na	na	na	pseudo (SeD_A4893)
4271	39	4510	31	8	none
4275.5	4.5	4514.5	4.5	0	yjiG-yjiJ
4298	22.5	4533	18.5	4	tsr
4316	18	4551	18	0	diguanylate cyclase
4337	21	4581	30	-9	yjjK
average	21.21	---	---	---	---
std dev	20.38	---	---	---	---

[a] [Column 1, boxed cells with solid lines] Genes in S. Enteritidis with ORFs containing a deletion or a change in termination codon; [Shaded region across all columns] region of inversion between *S. enteritidis* and *S. typhimurium genomes;* [Boxed rows across all columns] regions that have an offset of 5 genes or greater between S. Enteritidis and S. Typhimurium genomes, which are further explained in text.

[b] Numbers ending in "0.5" indicate SNPs located in an intergenic region. Intervals between genes and intergenic regions are calculated based on the presence of one or more SNPs. Positive values indicate regions where SEN has more genes than STM and negative values indicate regions where STM has more genes than SEN. Only absolute values are used to calculate average difference in alignment of genes between SEN and STM (column 5).

[c] Gene order is based on annotation of the *S. enteritidis* PT4 reference genome (Refseq: NC_011294)

[d] na, not applied due to annotation artifact from differences in rrn numbering or because the gene is absent in *S*. Typhimurium LT2 (Refseq: NC_003197).
